# Intermediates in the cation reactions in solution probed by an *in situ* surface enhanced Raman scattering method

**DOI:** 10.1038/srep13759

**Published:** 2015-09-03

**Authors:** Chih-Shan Tan, Hung-Ying Chen, Hsueh-Szu Chen, Shangjr Gwo, Lih-Juann Chen

**Affiliations:** 1 Department of Materials Science and Engineering, National Tsing Hua University, Hsinchu 30013, Taiwan; 2 Department of Physics, National Tsing Hua University, Hsinchu 30013, Taiwan

## Abstract

For chemical reactions in liquid state, such as catalysis, understanding of dynamical changes is conducive to practical applications. Solvation of copper salts in aqueous solution has implications for life, the environment, and industry. In an ongoing research, the question arises that why the color of aqueous CuCl_2_ solution changes with solution concentration? In this work, we have developed a convenient and efficient *in situ* surface enhanced Raman scattering technique to probe the presence of many intermediates, some of them are responsible for color change, in crystallization of aqueous copper chloride solution. The versatility of the novel technique was confirmed in the identification of five intermediates states in the transition from CdS to MoS_2_ nanowires in solution. The facile *in situ* method is expected to be widely applicable in probing intermediate states in a variety of chemical reactions in solution.

In an investigation of growing nanowires through sequential cation exchange^1^, it was noticed that the color of aqueous CuCl_2_ solution changes from light blue green to turquoise, green then dark green upon the increase in solution concentration. The natural question is what causes the change in color. Copper chloride is widely used for sterilizing bacteria in swimming pool^2^. In fact, the light blue green color of swimming pool water can often be attributed to the presence of Cu^2+^. In addition, copper chloride is a frequent oxidizing agent compound usually used for oxidizing phenol in chemical industry^3^. For electroless copper plating^4^, the research on intermediates is also critical for the improvement of the manufacturing process. Solvation of copper salts in aqueous solution has implications for life, the environment, and industry. In view of its importance in practical applications on top of the curiosity about the cause of a commonly observed physical phenomenon, we have carried out an investigation on the changes in aqueous CuCl_2_ solution upon the variation in solute concentration. A convenient and efficient *in situ* SERS technique was developed and the presence of many intermediates, some of them are responsible for color change, was readily detected. It is further revealed that intermediates undergo changes even under supersaturated conditions.

For chemical reactions in liquid state, understanding of dynamical changes is conducive to practical applications. As a prime example, research into catalysis, which is critically important in the production of most industrially important chemicals, is based on the reach to transition state with less energy or forming intermediates not produced naturally in the presence of catalysts. Among various transition metal catalysts, copper is considered to be most prominent and promising because of its versatility, low cost, and low toxicity^5^. In addition, copper is one of the most common transition elements in biological systems, a normal human can have 80 to 120 mg in their body^6^, and copper is present in a large number of enzymes, many involved in electron transfer, activation of oxygen, and oxygen transport^7^,^8^. Crichton^8^ showed that the Cu (II) four-coordinate complexes are of square planar and the Cu (I) four-coordinate complexes are of tetrahedral structure. Cu (II) is a transition metal ion with d^9^ (t_2g_^6^e_g_^3^) configuration and have significant Jahn-Teller effects. Identification of by-products^9^,^10^ and intermediates^11^,^12^,^13^ is an crucial step for providing complete picture of reaction pathways. *In situ* study of reactions provides a unique view of dynamic reactions in real time as they occur^14^,^15^,^16^,^17^,^18^. In the present study, a facile *in situ* surface-enhanced Raman scattering (SERS) technique was developed for the investigation of dynamical changes in copper chloride dihydrate crystallization process in CuCl_2_ aqueous solution. The intermediates in the crystallization of CuCl_2_ in water are clearly identified. The SERS substrates used are gold nanoparticles with two different average sizes on Si and PI film substrates. Copper chloride aqueous solution drop was first dripped on SERS substrates with a micropipette. *In situ* SERS observation was then carried out to detect the intermediates of Cu^2+^, Cl^−^ , and H_2_O during dynamic change from aqueous states to solid state. Crystallization was found to undergo different vibrational energy state changes before and after supersaturation in ionic solution. Here we show a facile method to investigate the dynamic change of bonding of transition metal ions, Cu^2+^, and emphasize the *in-situ* SERS observation as a facile technique for the observation of dynamic changes of intermediates in a variety of chemical reactions in solution. The versatility of the novel technique was confirmed in the identification of five intermediates states in the transition from CdS to MoS_2_ nanowires in solution.

SERS was first discovered in 1974^19^, Fleishman *et al*. utilize noble-metal surface to enhance the intensity and sensitivity of Raman spectroscopy. To increase sensitivity level and the applicability of SERS, a variety of nanostructures on surfaces with different morphologies^20^,^21^ were fabricated. In general, the sensitivity level critical to the enhancement behavior of SERS can be described by the enhancement factor (EF). The maximum SERS EF is a maximum localized charge density wave amplified on a specific positions (hot-spot) and the average SERS EF is an average charge density wave amplified on the surface. When the value of average SERS EF is as large as 10^7^–10^8^, it can be considered as a functional SERS substrate and is capable of detecting single-molecule^22^,^23^. The implication is that such a substrate^24^ is useful for precise *in situ* study. The ability to enhance Raman spectrum is fundamentally correlated not only with the precise details of the structure on the surface but also with the metallic material properties.

Figure 1a shows the photographs of CuCl_2_•2(H_2_O) (Alfa Asear, 99.999%) aqueous solutions with different mole concentrations, from left to right are pure water, solutions of 0.5 M, 0.75 M, 1 M, 2.5 M, 5 M, 7.5 M, 10 M, 12.5 M, 15 M and crystalline CuCl_2_•2(H_2_O). As shown in Fig. 1a, the solution color changes with concentration and if the concentration is higher than 10 M copper chloride dihydrate, it becomes supersaturated. An intriguing question is why the color of solution changes drastically from light blue to dark green with the increase in copper chloride concentration from 0.5 M to 7.5 M. Figure 1b shows UV/Visible transmittance spectra for copper chloride aqueous solutions (0.5 M, 0.75 M, 1 M, 2.5 M, 5 M, 7.5 M, 10 M, 12.5 M, and 15 M). It is apparent that the electronic states change continuously with the increase in copper chloride mole concentrations. The transmittance peaks change from 482 nm with broad full width at half maximum (FWHM) 176.6 nm at 0.5 M to 532 nm with broad FWHM 34.9 nm at 15 M. Interestingly, even under supersaturation conditions (10 M, 12.5 M, and 15 M), the wavelength, intensity, and FWHM of the absorption peaks still exhibit changes. It can be inferred that even when the solution is supersaturated, the addition of more solute (CuCl_2_) to the solution (CuCl_2_ aqueous solutions) still leads to the change in electronic states of the solution. Figure 1c shows the SERS spectra of copper chloride aqueous solutions (0.5 M, 0.75 M, 1 M, 2.5 M, 5 M, 7.5 M, 10 M, 12.5 M, and 15 M. It is seen that when concentration changes from 1 M to 2.5 M, a small peak at 289 cm^−1^ emerges. It is a bond between Cu^2+^ and Cl^1−^ and the presence of the bonds is the reason for solution color changes from blue (482 nm) to turquoise (504 nm)^24^. After the concentration is raised to 15 M, 214 cm^−1^ and 406 cm^−1^ peaks emerge indicating the formation of crystalline CuCl_2_•2(H_2_O) in solution^24^.

The intermediate states of copper chloride aqueous solutions have been investigated with density functional theory, ab initio methods, extended X-ray absorption fine structure (EXAFS), Minuit X-ray absorption near-edge structure (MXAN) and UV-vis-near IR optical absorption spectroscopy previously^25^,^26^,^27^,^28^. To capture the dynamic reaction of those Cu^2+^ and Cl^−^ during the concentration change from 1 M copper chloride aqueous solution to higher concentration, supersaturation and condensation, a drop of 1 M copper chloride aqueous solution was dispensed on the SERS substrate. The solution gradually evaporates at room temperature and eventually becomes crystalline **CuCl**_**2**_**•2(H**_**2**_**O)**. The total evaporation process takes about 20 min and allows *in-situ* Raman measurements (514 nm, green laser) at 20 s interval. The main changes were detected to occur at about 920 s to 1200 s (Fig. 2). In the beginning^24^ (920 s), the total solution is full of Cu(H_2_O)_6_^2+^ (Supplementary Fig. S7, stage **I**), as the times go by, a broad 289 cm^−1^ peak was generated (980 s), indicating the presence of a strong band of Cu-Cl bonds^24^. It is likely that that there is a complex mixture of CuCl^+^, CuCl_2_, CuCl_3_^−^, and CuCl4^2−^25. The complex mixtures (Supplementary Fig. S7, stage **II**) contribute to a broadened 289 cm^−1^ peak at 980 s and change to CuCl_4_^2−^ and CuCl_2_•2(H_2_O) at 1040 s (Supplementary Fig. S7, stage **III**). The 108 cm^−1^ peak indicates a long bond stretching mode for linear Cl-Cu-Cl in CuCl_2_•2 (H_2_O), the 214 cm^−1^ peak is ascribed to a Cu-Cl bond in CuCl_2_•2 (H_2_O) (a_g_), and the 406 cm^−1^ peak corresponds to Cu-O bond in CuCl_2_•2 (H_2_O). The 188 cm^−1^ peak indicates Cu-Cl bond in CuCl_4_^2-^ (a_g_) and 289 cm^−1^ peak corresponds to Cu-Cl bond in CuCl_4_^2–^ (b_2g_). At 1040 s there is a 188 cm^−1^ peak indicating the presence of Cu-Cl bond in the tetrahedral structure CuCl_4_^2−^ (a_g_) (Supplementary Fig. S7, stage **III**). From 1040 s to 1080 s, the Cu^2+^ sustains stereoisomer compounds change with the same surrounding Cl^−^, as the same CuCl_4_^2−^ compound but with the structural change from tetrahedral to square planar. At 1080 s, the compound transforms to CuCl_2_•2 (H_2_O) as the reaction is completed (Supplementary Fig. S7, stage **IV**).

In Fig. 3, the dynamic changes of crystallization from 1 M copper chloride aqueous solution is captured with different wavelength (632.8 nm, red) laser pump. The difference from changes shown in Fig. 2 is the additional observation of short duration vibrational states: 547 cm^−1^, 468 cm^−1^ and 568 cm^−1^. The three vibrational states can further reveal the mechanism of CuCl_2_•2 (H_2_O) crystallization in supersaturated solution. Peak at 547 cm^−1^ indicates that there is a Cu-O bond and the bond stretching vibration is like a dicopper(III) complex [Cu_2_(μ-O)_2_(Et_3_CY)_2_](SbF_6_)_2_^29^ at 290 s. At 320 s, peak at 468 cm^−1^ indicates a Cu-O bond^30^ and peak at 568 cm^−1^ indicating Cu-O bond like in CuO thin film^31^. The peaks not obvious in Fig. 2 but evident in Fig. 4 are corresponding to those vibration states more sensitive to the probing of 632.8 nm laser irradiation than that of 514 nm laser. It indicates that during crystallization, oxygen atoms bond with different angles and lengths to Cu (II) atom in CuCl^2−^.

Figure 4 summarizes vibration modes detected during the drying process of copper chloride aqueous solution (1 M) by real time SERS. SERS peaks appear at different times and concentrations can be differentiated into four stages. Stage I is the initial state with low solute concentrations. In this stage the bonds with Cu^2+^ are too weak for detection although the presence of Cu(H_2_O)_6_^2+^ was detected by optical absorption spectroscopy reported in ref. 32. Stage II is signaled by the appearance of 289 cm^−1^ peak indicating the solute concentration is high enough so that the corresponding bonds are detectable by SERS. Stage III represents supersaturation stage with the appearance of vibration mode of 214 cm^−1^ as seen in Fig. 1 (c). Other modes, such as 108 cm^−1^, 188 cm^−1^, 289 cm^−1^ and 406 cm^−1^ are also detected under green laser irradiation. Additionally, transition states exhibited by 547 cm^−1^, 468 cm^−1^, and 568 cm^−1^ peaks appear at different time and are only evident under red light laser irradiation. In the final stage (stage IV), copper chloride aqueous solution is crystallized to form CuCl_2_•2 (H_2_O) and CuCl_4_^2−^ (square planar) compounds and SERS peaks would no longer change with time.

Figure 5 shows the real time change in SERS signals in the transition of a single CdS to MoS_2_ nanowires by cation exchange obtained with *in situ* observation at room temperature (Supplementary Fig. S8a). The results represent the first *in situ* observation of cation exchange^32^,^33^ process. In the SERS spectra, in the beginning (0 s), the 299 cm^−1^ peak corresponds to first order longitudinal optical mode (1LO) of CdS. At 340 s, the E_2g_^1^ (391 cm^−1^) and A_1g_ (399 cm^−1^) signals of 2-D MoS_2_ structure emerge (Supplementary Fig. S9d). Overall, five intermediate states emitting 454 cm^−1^, 470 cm^−1^, 638 cm^−1^, 750 cm^−1^, and 798 cm^−1^ signals were identified. Both the 454 cm^−1^ and 470 cm^−1^ peaks arise after 40 s reaction and at 100 s reach the maximum intensities (Supplementary Fig. S8b). The two peaks can be ascribed to two phonon vibration mode, 2LA(M)^34^ and A_2u_^35^. At 100 s, 750 cm^−1^ peak indicates the presence of 2E_2g_^1^ vibration mode^36^. The 798 cm^−1^ and 638 cm^−1^ peaks appear at 220 s and 260 s and represent Mo-O-Mo vibrations^37^ and A_1g_(M) + LA(M) mode^34^, respectively. The *in situ* data indicate that in the transition from CdS to MoS_2_ by cation exchange at room temperature, five intermediate states are detected by the novel method. In addition, the differences between in plane vibration (E_2g_^1^) and out of plane vibration (A_1g_) of MoS_2_ during cation exchange (Supplementary Fig. S9) vary with the time and are 10 cm^−1^ (120 s), 11 cm^−1^ (200 s), 10 cm^−1^ (280 s), and 8 cm^−1^ (340 s) perhaps indicating the dynamical change in strain^36^.

514 nm laser (green laser) with 5 nm FWHM was also used as a light source to pump the CdS NW on SERS substrate. The green laser tends to excite CdS NW to emit green light since the band gap of CdS is around 2.4 eV (~516 nm). As a result, the Raman signals are obscured. It indicates that the method is constrained by the type of laser probe used as well as the band gap of the semiconductor nanowire.

In summary, we have developed a convenient and effective *in situ* SERS method to probe the intermediate states in the crystallization of copper chloride aqueous solution. From optical transmittance measurement and Raman spectroscopy, it is elucidated that the appearance of 289 cm^−1^ peak, corresponding to various Cu-Cl bonds, accompanies the change of solution color from light blue to green. Several intermediate states are disclosed by the *in situ* SERS under green and red laser irradiations. It is further revealed that intermediates undergo changes even under supersaturated condition. The versatility of the novel technique was confirmed in the identification of five intermediates states in the transition from CdS to MoS_2_ nanowires in solution. The facile *in situ* SERS technique is expected to be widely applicable for investigating the intermediate states in a variety of chemical reactions, which are of both fundamental and practical importance.

## Additional Information

**How to cite this article**: Tan, C.-S. *et al*. Intermediates in the cation reactions in solution probed by an *in situ* surface enhanced Raman scattering method. *Sci. Rep*. **5**, 13759; doi: 10.1038/srep13759 (2015).

## Supplementary Material

Supplementary Information

## Figures and Tables

**Figure 1:**
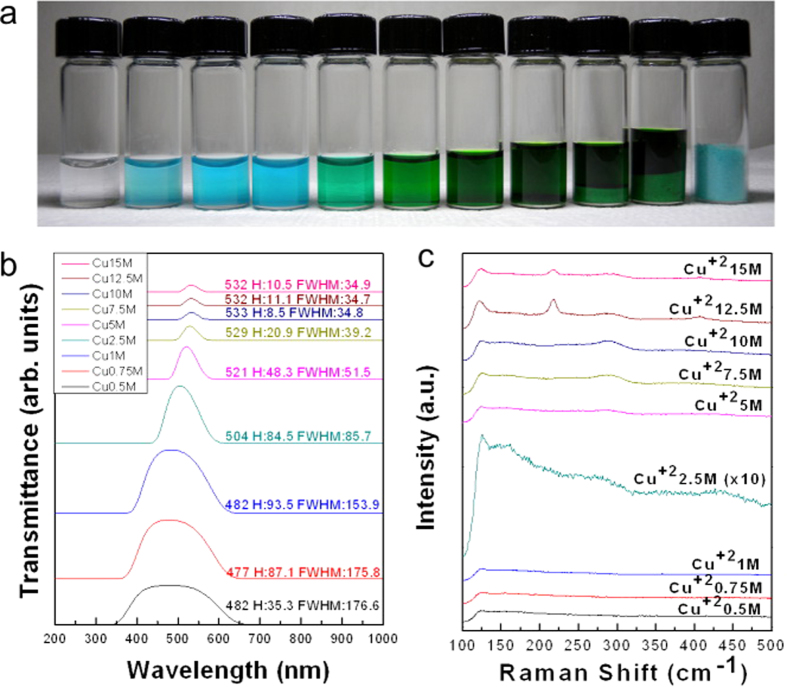
Copper chloride aqueous solutions with different mole concentrations. (**a**) From left to right, pure water, solutions of copper chloride 0.5 M, 0.75 M, 1 M, 2.5 M, 5 M, 7.5 M, 10 M, 12.5 M, 15 M, and copper chloride dihydrate. (**b**) UV/Visible transmittance spectra for probing the electronic structures of copper chloride solutions with different concentrations, and H is the transmittance in arbitrary unit. (**c**) SERS spectra for probing the vibrational structures of copper chloride solutions with different concentrations.

**Figure 2:**
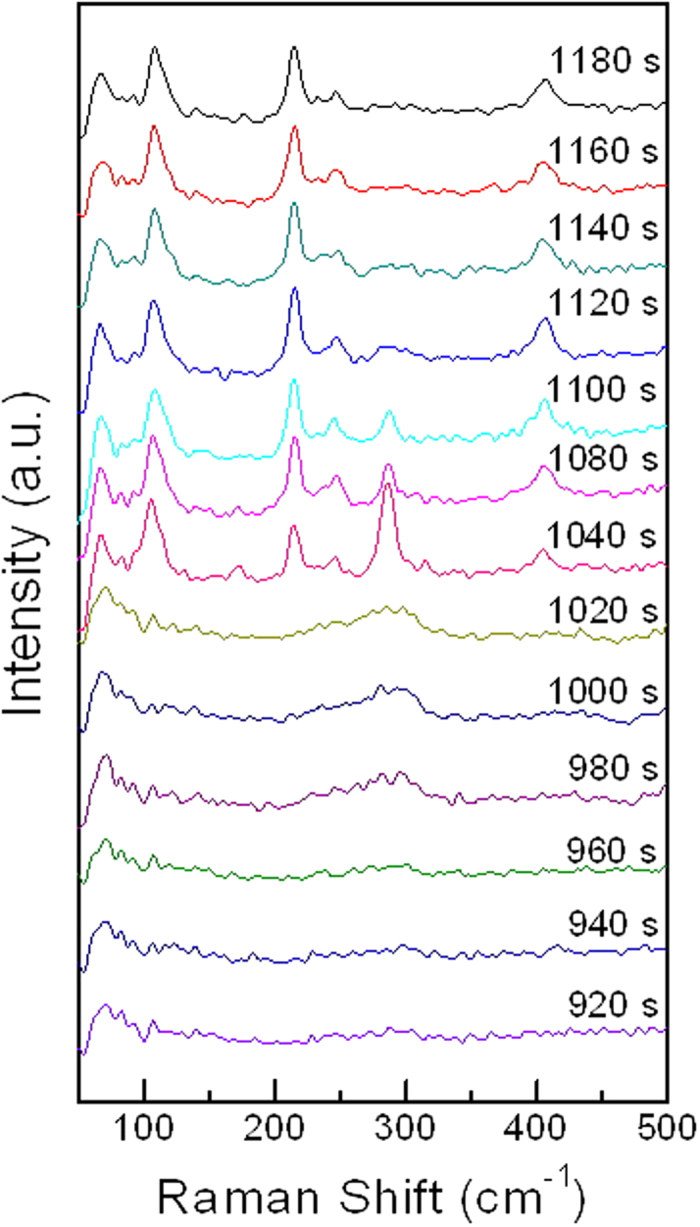
Time dependent *in situ* SERS (with 2 mW, 514 nm laser) spectra of crystallization of 1 M copper chloride aqueous solution to copper chloride dihydrate.

**Figure 3:**
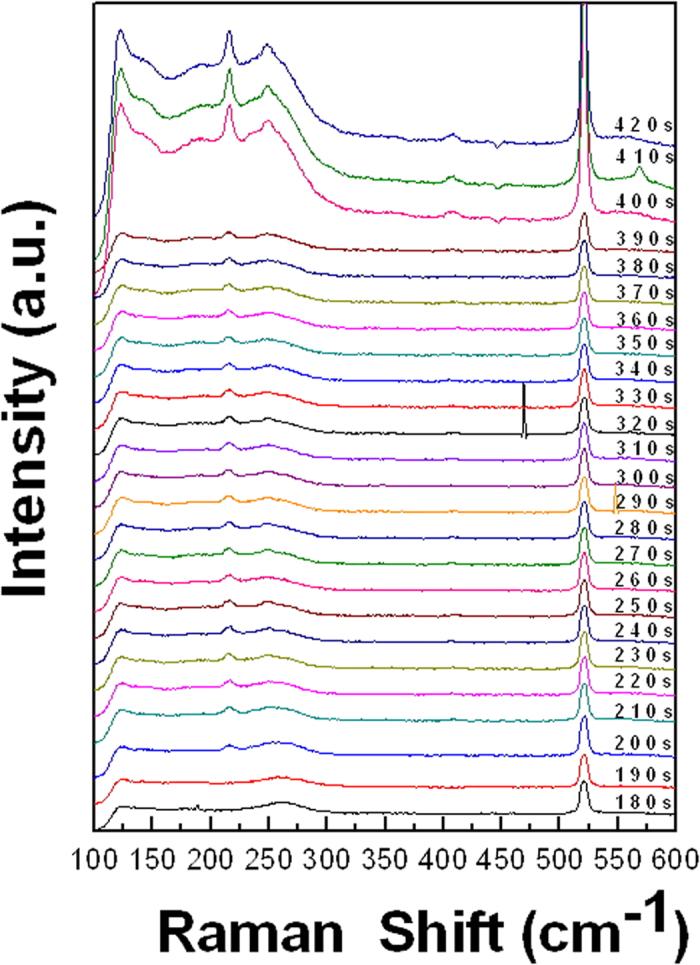
Time dependent *in situ* SERS (with 17 mW, 632.8 nm laser) spectra of crystallization of 1 M copper chloride aqueous solution to copper chloride dihydrate.

**Figure 4:**
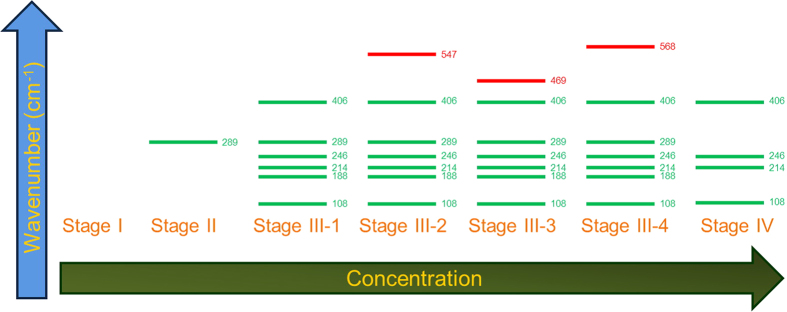
Illustration of copper chloride aqueous solution crystallization process with four stages. The green and red lines represent states revealed by the probing of 514 nm (green) and 632.8 nm (red) lasers, respectively.

**Figure 5:**
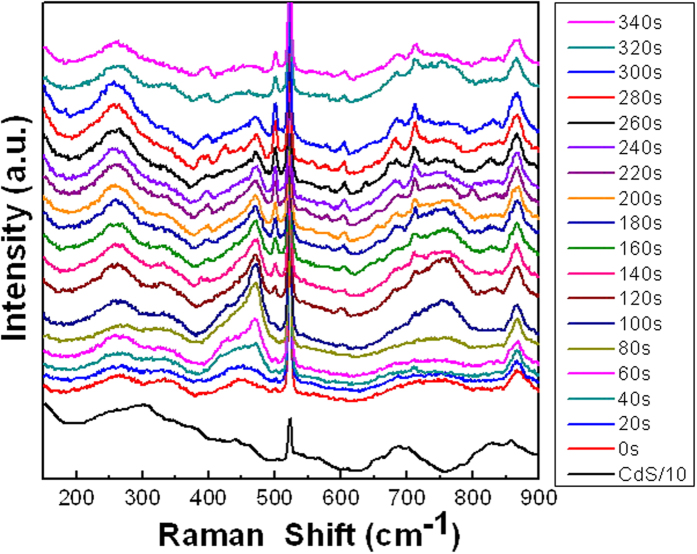
Time dependent *in situ* SERS (with 17 mW, 632.8 nm laser) spectra obtained for cation exchange from a single CdS to MoS_2_ nanowire in 0.1 M Mo ion solution.
