# Supplementary material for: Intermediates in the cation reactions in solution probed by an in situ surface enhanced Raman scattering method
Source: Sci Rep. 2015 Sep 3;5:13759. doi: 10.1038/srep13759 (PMC4558718; doi:10.1038/srep13759)
Supplement: Supplementary Information [file srep13759-s1.pdf]

Supplementary Materials for

Intermediates in cation reactions in solution probed by an in situ surface enhanced Raman scattering method

Chih-Shan Tan[1], Hung-Ying Chen[2], Hsueh-Szu Chen[2], Shangjr Gwo[2], Lih-Juann Chen[1,]*

Affiliations:

[1]Department of Materials Science and Engineering; and

[2]Department of Physics, National Tsing Hua University, Hsinchu 30013, Taiwan

*Correspondence to: ljchen@mx.nthu.edu.tw

This file includes:

Materials and Methods

Figs. S1 to S8

Materials and Methods

Gold nanoparticles synthesis:

For preparing good SERS substrates, we develop a facile method to fabricate gold nano particles with two sizes (10 and 100 nm) on Si substrate and achieve a high EF_{SERS} value ($>10^7$). The low temperature processing (with a maximum temperature of 250 ^oC) facilitates the fabrication of SERS structure on flexible polyimide (PI) film for possible applications. According to the previous research [22], SERS has a strong connection with plasmonic phenomenon which is an electromagnetic wave induced charge density variation on the noble-metal nanostructure and dielectric surface. Gold has a very good quality factor (Q) of localized surface plasmon resonance on an Au nanoparticle (NP)/ air surface for incidence of light with wavelengths beyond ~600 nm[21] (red and near IR). With Raman mapping capability, the localized hot spots on the SERS substrate can be distinguished. From the analysis of the density of hot spot, we can clearly correlate surface plasmon with surface-enhanced Raman scattering behavior. A schematic illustration is shown in Fig. S1.

Our SERS substrate is prepared by dispersing Au NPs on Si (100) wafer with a two-step process: e-gun evaporation of 10 nm Au film from gold slug (Gredmann, 99.99%) and thermal evaporation of gold acetate (Alfa Aesar, 99.9%) in a three zone furnace (Fig. S2a). Electron beam evaporation was conducted at a pressure better than $5x10^{-6}$ torr. The two-step process can produce two kinds of Au NPs on Si (100) wafer with an average diameter around 10 nm and 100 nm. Fig. S2b illustrates the process of heating gold acetate powder at 300 ^{o}C in a carrier gas with a mixture of 100 sccm and 50 sccm H_2 at a pressure of 3 torr. The gold acetate powder evaporated in the furnace and the vapors were reduced to gold by reacting with H_2. Si (100) and Au (10nm)/ Si (100) wafers were placed at 225 ^{o}C, 250 ^{o}C, and 275 ^{o}C regions for receiving the Au deposition. Six kinds of substrates with Au NPs on Si (100) wafer were prepared as shown in Figs. S2c-h. For Au NPs formed on 10 nm Au/Si(001) wafers at 250 ^{o}C (Fig. S2c), the SERS with R6G (10^{-5}M) incubation was found to be most prominent. From the measurement, $EF_{SERS} > 10^7$ was obtained, which is adequate for the ultra-sensitivity Raman spectrum detection. In Fig. S3, we show that with the incubation of 10^{-8}M R6G, the R6G peak is detected indicating the capability of detecting single molecular R6G. With the excellent sensitivity, the in situ SERS observation can detect intermediates of single-molecule scale in chemical reaction solution.

Raman measurement and mapping:

Horiba Jobin Yvon, LABRAM HR 800 UV was used for Raman measurement. Fig. 4a shows the SERS spectrum for 10^{-7} M R6G solution. Red framed area in Fig. S4b was used for 3D (Fig. S3c) and 2D (Fig. S3d) SERS mapping studies. From OM image shown in Fig. S3b we can identify the localize hot-spot positions. The dark spots (Au NPs) in OM photo correspond to the localized large charge density wave amplification positions in Fig. S3b. In Fig. S3c, we map 2.7 x 2.7 μm^2 area with 30 x 30 points, and use Raman intensity from each point deviated from background Raman intensity (choosing S4 850-900 cm^{-1} intensity as background). 3D and 2D local intensity/background intensity ratio mappings are shown in Figs. S3c and S3d, respectively. The intensity ratio increases from blue, green, yellow, to red, and there are four highest hot-spots in this mapping with red color. Fig. S3c is the 3D color mapping by transforming the 2D mapping data to a more clearly visible mapping. Localized hot-spots appear as peaks with different attitudes. Figure S3e is a histogram of the local intensity/background intensity ratio from the 900 mapping data points. The intensity ratios of most spots are about 5-10 (around 600 points. The ratios of the other about 50 points are lower than 5-10, and while more than 250 points are higher than 5-10. The highest energy ratio points can attend 34 around 5-7 points in the area. The data indicate that although the surface plasmon has different abilities to enhance

on localized positions, their energy ratios largely follow Gaussian distribution and higher energy region is more than 5 times than that of lower energy region at most of the locations

CdS NW synthesis: CdS NWs were grown with the procedures described in ref. 1. After growth, we place them on SERS substrate.

***In situ* cation exchange**

An ethylene glycol (99.5%, Sigma-Aldrich) droplet containing 0.1 M Molybdenum(V) chloride (Alfa Aesar, 99.6%) is dispensed on CdS NWs/SERS substrate at room temperature with a micropipette for cation exchange. The in situ SERS data were obtained with a Horiba Jobin Yvon, LABRAM HR 800 UV system at 20 s interval.

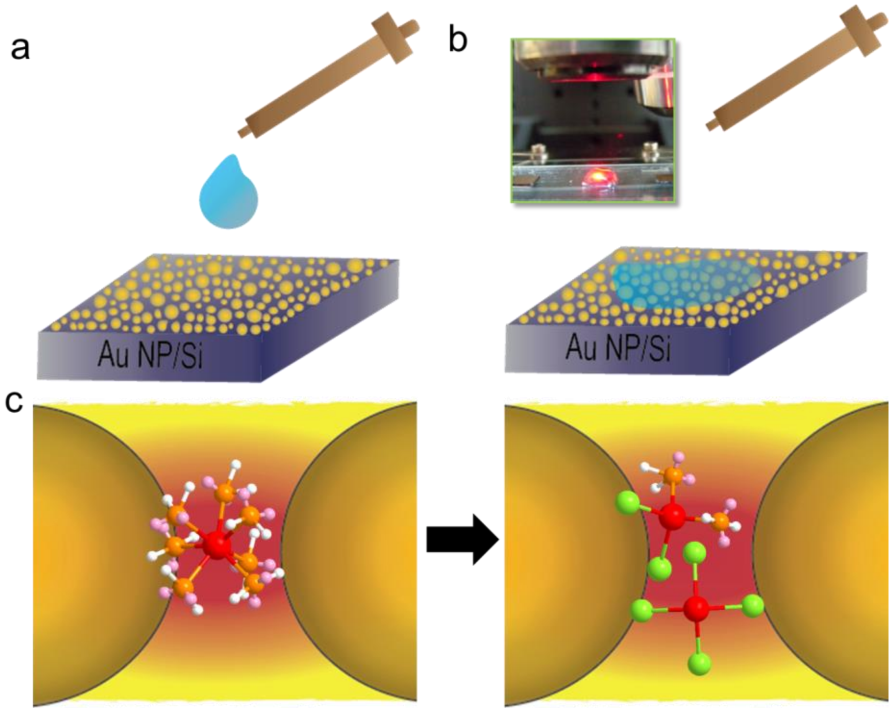

Fig. S1.

Illustration of the *in situ* SERS method. (a) The dispensation of a liquid droplet from a micropipette. (b) The droplet spreads on SERS substrate. (c) The molecular transitions in the hot spot region.

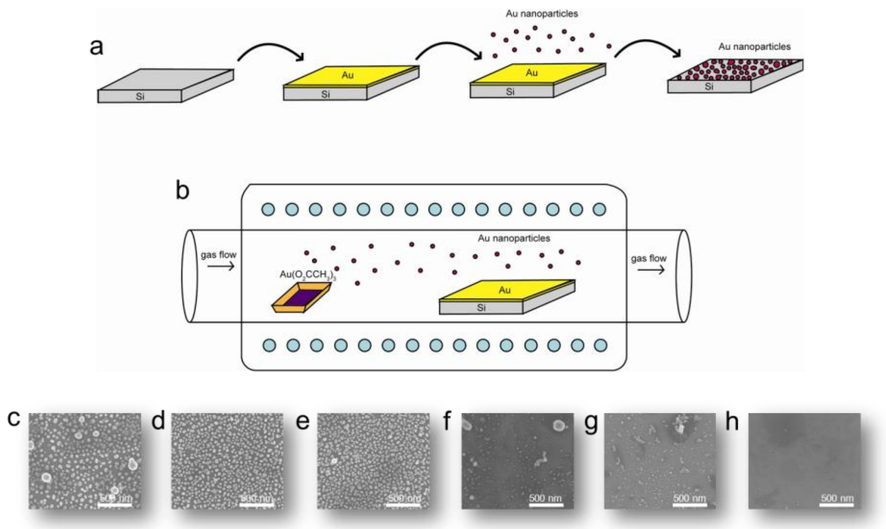

Fig. S2.

Illustration of the preparation of SERS. (**a**) Two-step process to sysnthsize two sizes of Au nanoparticles on Si wafer. (**b**) The process for evaporating gold acetate powder to Au (10 nm)/Si wafer in a three zone furnace. Evaporation of gold acetate powder to Au (10nm)/Si at (c) 275 ^{o}C, (d) 250 ^{o}C, (e) 225 ^{o}C regions. Evaporation of gold acetate powder to Si at (f) 275 ^{o}C, (g) 250 ^{o}C and (h) 225 ^{o}C regions.

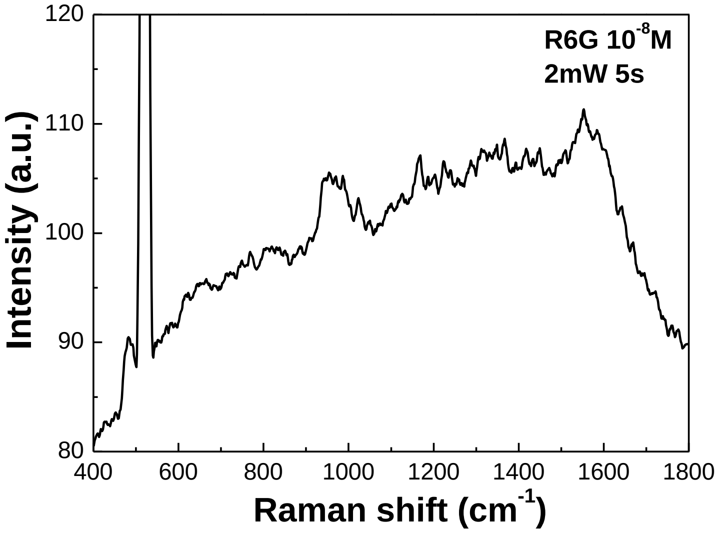

Fig. S3

Distinguishable signals are still evident for dispensation of a 10^{-8}M R6G droplet on SERS substrate.

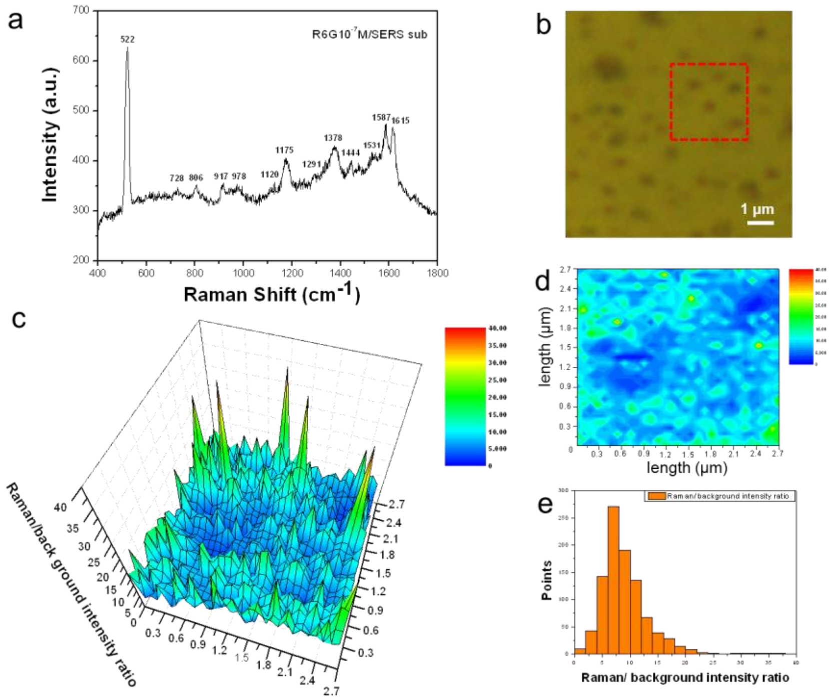

Fig. S4

Enhancement factor and Raman mapping. (**a**) Raman spectrum for 10^{-7} M R6G solution on SERS substrate. (**b**) OM photo of the SERS substrate with the red frame outlines Raman mapping area. (**c**) 3D Raman mapping, the sharp hills correspond to hot spots. (**d**) 2D Raman mapping figure. (**e**) Intensity distribution of 900 Raman mapping points.

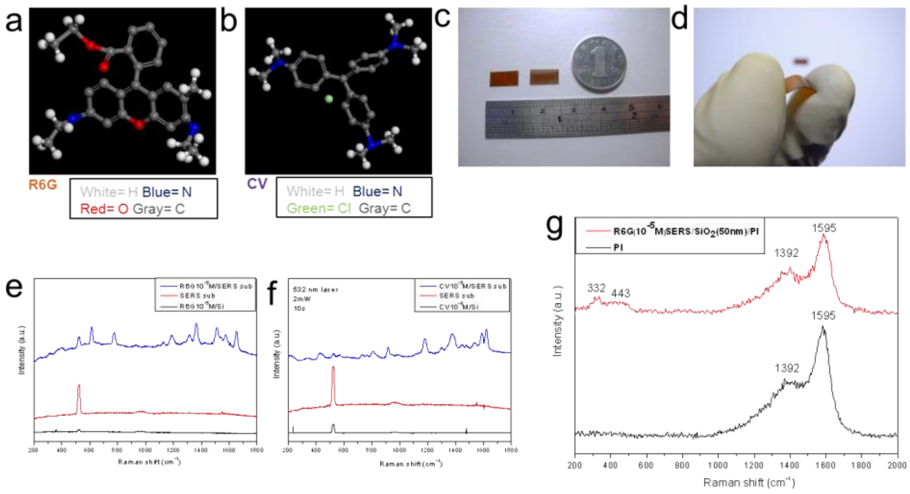

Fig. S5

Molecules of Rhodamine 6G (R6G) and Cryatal violet (CV) laser dyes and the flexible SERS substrate. (**a**) R6G molecule. (**b**) CV molecule. (**c**) The SERS substrate prepared on PI film. (**d**) Demonstration of the flexibility of the flexible SERS substrate. (**e**) SERS effect for R6G solution (10^{-5} M) on the SERS structure prepared on Si substrate and pure Si substrate. (**f**) SERS effect for CV solution (10^{-5} M) on the SERS structure prepared on Si substrate and pure Si substrate. (**g**) The SERS structure made on PI film and still showing SERS effect with R6G solution (10^{-5} M) for the enhancement of 332 and 443 cm^{-1} peaks.

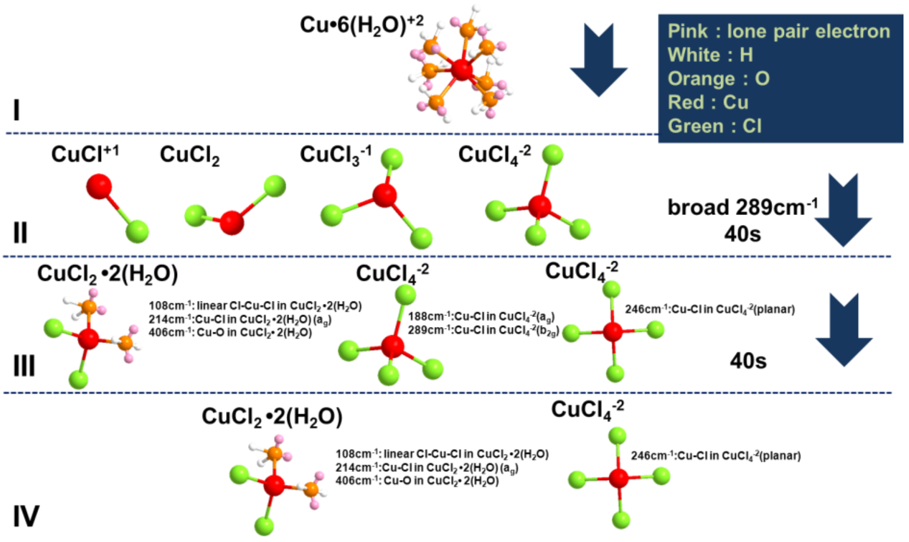

Fig. S6

Illustrations of copper chloride aqueous solution transformation to copper chloride dihydrate reaction. The reaction is divided to four stages. In stage I, the aqueous solution is full of Cu•6(H_2O) $^{+2}$. In stage II, the Cu^{+2} is surrounded by Cl^- with four kinds of transition states: $CuCl^{+1}$, CuCl, $CuCl_3^{-1}$, and $CuCl_4^{-2}$. In stage III, $CuCl_2$•2(H_2O) and $CuCl_4^{-2}$ coexist, but $CuCl_4^{-2}$ have two isomeric structures: tetrahedral and square planar. In stage IV, $CuCl_2$•2(H_2O) coexists with $CuCl_4^{-2}$ (square planar).

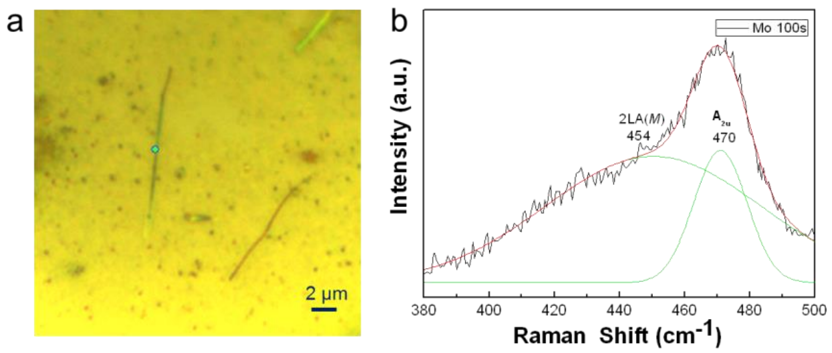

Fig. S7

Optical and SERS data of *in situ* cation exchange observation by SERS. (**a**) Optical image of a CdS nanowire on SERS substrate for cation exchange to MoS_2 for in situ SERS observation. The blue dot indicates the spot probed with the laser irradiation. (**b**) Intermediate states revealed by curve fitting of SERS spectrum obtained for the transition from CdS to MoS_2 at 100 s.

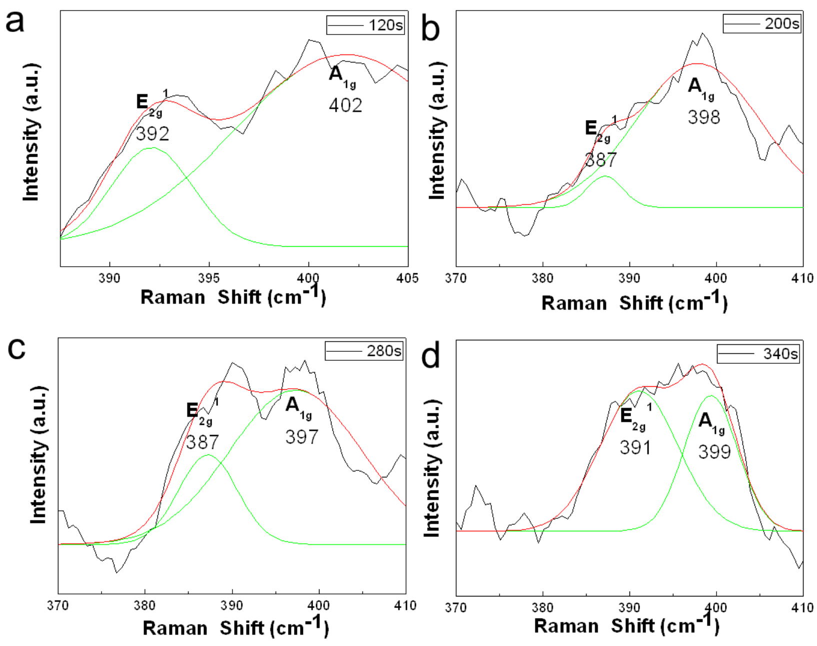

Fig. S8

The MoS_2 E_{2g}^1 and A_{1g} peak fitting of SERS data obtained by *in situ* probing of cation exchange at (**a**)120 s, (**b**) 200 s, (**c**) 280 s and (**d**) 340 s.
